# Chronological Age in Different Bone Development Stages: A Retrospective Comparative Study

**DOI:** 10.3390/children8020142

**Published:** 2021-02-13

**Authors:** Abel Emanuel Moca, Luminița Ligia Vaida, Rahela Tabita Moca, Anamaria Violeta Țuțuianu, Călin Florin Bochiș, Sergiu Alin Bochiș, Diana Carina Iovanovici, Bianca Maria Negruțiu

**Affiliations:** 1 Department of Dentistry, Faculty of Medicine and Pharmacy, University of Oradea, 1 Universității Street, 410087 Oradea, Romania; abelmoca@yahoo.com (A.E.M.); ligia_vaida@yahoo.com (L.L.V.); bochis.alin@yahoo.com (S.A.B.); biancastanis@yahoo.com (B.M.N.); 2 Clinical Emergency County Hospital Oradea, 37 Republicii Street, 410167 Oradea, Romania; rahelamoca@gmail.com; 3 Clinical Emergency Municipal Hospital Timișoara, 1 Hector Street, 300041 Timișoara, Romania; 4 Faculty of Medicine, University of Medicine and Pharmacy “Victor Babeș”, 2 Eftimie Murgu Square, 300041 Timișoara, Romania; diana_iovanovici@yahoo.com

**Keywords:** chronological age, skeletal maturity, Cervical Vertebral Maturation

## Abstract

The assessment of an individual’s development by investigating the skeletal maturity is of much use in various medical fields. Skeletal maturity can be estimated by evaluating the morphology of the cervical vertebrae. The aim of this study was to conduct comparisons of the chronological age in different bone development stages. The retrospective study was conducted based on lateral cephalometric radiographs belonging to patients with ages between 6 and 15.9 years, from Romania. For the assessment of skeletal maturity, the Cervical Vertebral Maturation (CVM) method was used. In total, 356 radiographs were selected, but after applying the exclusion criteria, 252 radiographs remained in the study (178 girls and 74 boys). Different mean chronological age values were obtained for the general sample, as well as for the two genders. The chronological age started to be significantly different at the CS4 stage. Patients with CS4, CS5, and CS6 stages had a significantly higher chronological age compared to patients with CS1, CS2, and CS3 stages. It was noted that patients with CS1 and CS2 stages were more frequently boys, while patients with the CS5 stage were more frequently girls.

## 1. Introduction

In medicine, age is essential for assessing the overall development of a patient. The chronological age, although easiest to determine if the child´s date of birth is known, often does not accurately reflect a patient´s development [1]. Various methods have been used to more precisely reproduce different developmental stages. These methods are based on determining the dental age [2] and skeletal age [3].

The assessment of skeletal maturity is useful in many fields, such as pediatrics, endocrinology [4], pediatric dentistry, and orthodontics [5]. In orthodontics, the degree of skeletal maturity influences the treatment planning and the optimal choice of treatment [6]. Hand-wrist radiographs have traditionally been used to estimate bone maturity [7], but skeletal age determination techniques based on the inspection of other bone structures have been suggested [8].

The radiological aspect of the cervical vertebrae can be used to estimate the degree of bone development. The method based on the investigation of cervical vertebrae has undergone several changes over time [9,10] and is currently known as the Cervical Vertebral Maturation (CVM) method. It involves the examination of cervical vertebrae 2, 3, and 4 on a lateral cephalometric radiograph [11]. Lateral cephalometric radiographs are necessary for establishing the diagnosis and treatment plan in orthodontics. Therefore, the assessment of skeletal maturity is possible, without any need for additional irradiation [12]. Further studies need to be conducted in order to find associations between age and CVM developmental stages.

The purpose of this study was to conduct comparisons of mean values of the chronological age in different skeletal developmental stages, for boys and girls, using the CVM method.

## 2. Materials and Methods

### 2.1. Sample Selection

This study is a retrospective and comparative radiographic study, performed on lateral cephalometric radiographs belonging to children form North-Western Romania. The lateral cephalometric radiographs were collected from three different dental private practices from the city of Oradea, Romania. The radiographs were previously used for diagnosis and treatment planning.

We included radiographs of children with ages between 6 and 15.9 years, radiographs of children for whom a signed consent form was obtained, radiographs available in a digital format, radiographs of patients with a known date of birth and known date of the radiograph, and radiographs of patients with a known gender.

Radiographs excluded from the study belonged to patients from other countries, patients with systemic diseases or genetic disorders that could impact the skeletal maturation, and patients that followed or were following an orthodontic treatment at the date when the radiographs were taken.

The selected lateral cephalometric radiographs were divided according to the gender of the patients. A total of 356 radiographs were initially selected, but after applying the exclusion criteria, only 252 were left in the study sample. The final sample consisted of 178 radiographs belonging to girls (70.6%) and 74 radiographs belonging to boys (29.4%).

### 2.2. Skeletal Maturity Assessment

For the assessment of skeletal maturity, the CVM method was used, as described by Baccetti et al. (2005). The CVM method consists of six different maturation stages (from CS1 to CS6), according to different morphological features of cervical vertebrae 2, 3, and 4. The inferior border of the three cervical vertebrae must be investigated, as well as the shape of the third and fourth cervical vertebrae [11].

**Figure 1:**
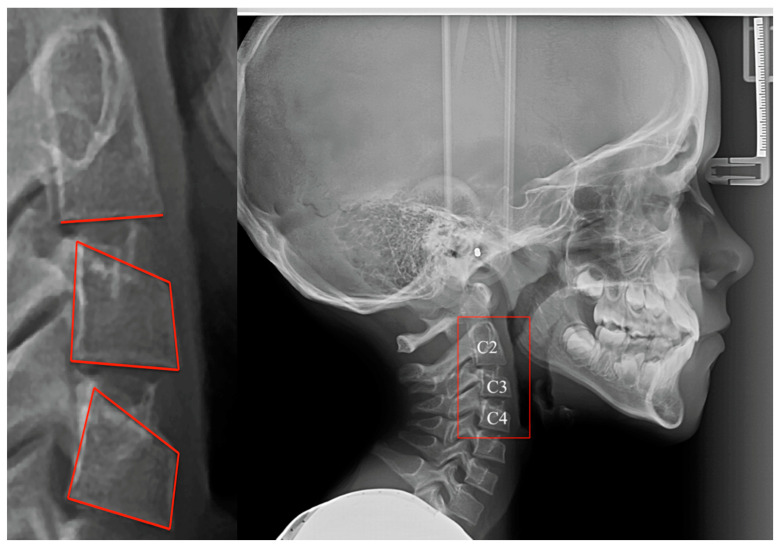
Lateral cephalometric radiograph with the 2, 3, and 4 cervical vertebrae highlighted.

The CVM method was applied on lateral cephalometric radiographs, available in a digital format, by examining the morphological changes of the cervical vertebrae and comparing them with the different developmental stages (Figure 1). In order to avoid inter-operator bias, the examination was performed by a single investigator (M.A.E.).

### 2.3. Statistical Analysis

The statistical analysis was performed by using IBM SPSS software, version 20 (IBM, Chicago, IL, USA). Quantitative variables were tested for a normal distribution by using the Shapiro–Wilk test and were expressed as the means with standard deviations, while categorical variables were expressed as counts or percentages.

The independent quantitative variables with a non-parametric distribution were tested by using a Mann–Whitney U test or Kruskal–Wallis H test. The independent quantitative variables with a parametric distribution were tested by using the One-Way ANOVA test. Categorical variables were tested by using Fisher´s Exact test, and Z tests with Bonferroni correction were performed in order to further detail the results. A post-hoc Tukey HSD test and Dunn–Bonferroni test were performed in order to detail the results obtained after testing the quantitative variables.

### 2.4. Ethical Considerations

The study was approved by the Research Ethics Committee of the University of Oradea (No.7/15.10.2020) and was conducted in accordance with the 1964 Declaration of Helsinki and its later amendments. All radiographs belonged to patients for whom an informed consent form was previously signed by the parents.

## 3. Results

The mean chronological age of the patients was 11.52 ± 2.23 years, with a median (interquartile range, IQR) value of 11.65 years. The minimum age was 6.2 years and the maximum age was 15.9 years. The data in Table 1 represent the comparison of the chronological age related to the gender, with the age distribution being non-parametric in both groups, according to the Shapiro–Wilk test. The Mann–Whitney U test shows that the differences between the groups were not significant.

Most of the patients were distributed in the CS4 and CS5 developmental stages and the fewest were distributed in the CS2 developmental stage (Table 2). The distribution of patients according to their gender and the CVM stage revealed significant differences between the investigated groups, and –Z test with Bonferroni correction showed that the patients with CS1 and CS2 developmental stages were more frequently boys, while patients with the CS5 developmental stage were more frequently girls (Table 3).

The data in Table 4 and Table 5 represent the comparison of the chronological age related to the CVM stages. The age distribution was non-parametric for patients with CS1 and CS2 stages, according to the Shapiro–Wilk test (*p* < 0.05). According to the Kruskal–Wallis H test, the differences were significant (*p* < 0.001), and the post-hoc tests showed the slow increase of the chronological age in relation to the increase of the CVM stage. The chronological age started to be significantly different at the CS4 stage. In the studied sample, the chronological age was not significantly different (*p* > 0.05) between patients with CS1, CS2, and CS3 stages. Patients with CS4, CS5, and CS6 stages had a significantly higher chronological age compared to patients with CS1, CS2, and CS3 stages, according to the post-hoc test (*p* < 0.01). Among patients with CS4, CS5, and CS6 stages, the chronological age was only significantly different between patients with CS4 and CS6 stages, and patients with CS6 stages had a significantly higher chronological age (*p* = 0.002).

The data in Table 6 and Table 7 represent the comparison of the chronological age related to the CVM stages for the girls sample, with results similar to the general sample, while the data in Table 8 and Table 9 represent the comparison of the chronological age related to the CVM stages for the boys sample, which showed that the age distribution was parametric, according to the Shapiro–Wilk test (*p* > 0.05). According to the One-Way ANOVA test, the differences were significant (*p* < 0.001).

## 4. Discussion

The usefulness of the CVM method for determining the skeletal age has been suggested by many authors. Gandini et al. (2006) highlighted not only the practicality of the method, but also the low radiation dose required by lateral cephalometric radiography [13]. The CVM method seems to be able to replace the hand and wrist radiography for the estimation of bone development and can be used with confidence for this purpose [14,15]. Moreover, lateral cephalometric radiographs can be used to establish various orthodontic diagnoses [12,16], as well as to assess the morphology of other bone structures in the craniofacial region [17]. Mandibular growth can also be safely and correctly assessed on lateral cephalometric radiographs [18]. They can even be used for an evaluation of the upper airways [19]. However, when vertebral anomalies are suspected, such as osseous torticollis, examinations such as 3D-CT may be required [20].

In this study, the CVM assessment was performed manually, by direct examination of the lateral cephalometric radiographs, but computerized methods for identifying CVM stages have been developed. Vaida et al. (2019) identified the CVM stages using OnyxCeph, which is computerized software, and correlated the skeletal age of the patients with the chronological age and dental age [21]. Certain smartphone applications that allow skeletal maturity assessment based on the vertebral morphology have also been developed [22].

Most authors have found correlations between CVM stages and the chronological age. In our study, we aimed to compare the chronological age in different CVM stages and no other correlations were explored. We wanted to discover whether important differences existed between the chronological ages of various CVM stages. However, mean values of the chronological age were obtained for each CVM stage. The comparisons were conducted for the entire sample, but also, separately, for girls and boys. It was observed that the chronological age started to be significantly different starting with the CS4 stage, for the general sample, as well as for boys and girls. In other populations, correlations have been found between the skeletal age and chronological age [23]. Safavi et al. (2015) reported a positive correlation between the chronological age and all of the CVM stages in a group of Iranian girls, highlighting a moderate correlation during the circumpubertal phase. The mean chronological ages obtained for the CS4 and CS5 stages in the Iranian sample are similar to those obtained for the girls in our study. The mean chronological age in CS4 and CS5 for the Iranian sample was 11.93 and 12.66 years, respectively, while for the girls in our study, the mean chronological age in CS4 and CS5 was 12 and 12.9 years, respectively (median values) [23]. Other authors have suggested that prepubertal skeletal development may be predicted in patients with an early stage of dentition [24,25].

Some authors suggest that the chronological age is not a reliable indicator for the assessment of skeletal maturity [26].

Skeletal development can also be compared or correlated with the dental age. Różyło-Kalinowska et al. (2011) found a moderate correlation between the stages of dental development and the stages of development of the cervical vertebrae, identifying faster skeletal development for the group of female patients [27]. Faster skeletal development in female patients was also observed in our sample. The mean chronological age of the girls was generally lower than the mean chronological age of the boys for each of the CVM stages. The girls in the CS3 stage, for example, had a mean chronological age of 10.16 years, while the boys had a mean chronological age of 11.01 years. The situation is consistent for all of the CVM stages. Other authors have identified a faster development of different bone structures in female patients. Maspero et al. (2020) concluded that the development of the maxillary sinuses in girls occurred earlier than in boys, but in both genders, the development overlapped with the peak of growth [28].

Chronological age determination based on the development of the cervical vertebrae can also be used when a child´s date of birth is unknown. Mishori R. (2019) described the case of a 17-year-old boy fleeing from Honduras to the United States of America, who was initially placed in an adult facility. He was later transferred to an age-appropriate facility, after a dental exam which revealed that he was only 16–17 years old. However, age determination based on imagistic methods can be inaccurate and should be adapted in different populations [29].

## 5. Conclusions

The distribution of patients according to their gender and CVM stage showed significant differences between the investigated groups. Patients with CS1 and CS2 developmental stages were more frequently boys, while patients with CS5 developmental stage were more frequently girls.

In the studied sample, the chronological age was not significantly different between patients with CS1, CS2, and CS3 stages. Patients with CS4, CS5, and CS6 stages had a significantly higher chronological age compared to patients with CS1, CS2, and CS3 stages. The various mean chronological ages started to be significantly different at the CS4 stage, but differences between stages were also identified.

## Figures and Tables

**Table 1:** Chronological age according to the gender.

Gender	Mean Age (Years) ± SD	Median (IQR)	Medium Rank	*p* *
Girls (*p* = 0.035 **)	11.535 ± 2.22	11.65 (10–13.025)	127.09	0.841
Boys (*p* = 0.029 **)	11.5 ± 2.29	11.65 (9.575–13.7)	125.07	

SD, standard deviation; IQR, interquartile range; * Mann–Whitney U Test; ** Shapiro–Wilk Test.

**Table 2:** Distribution of the patients according to the Cervical Vertebral Maturation (CVM) stage.

CVM Stage	No.	Percentage
CS1	38	15.1%
CS2	27	10.7%
CS3	35	13.9%
CS4	64	25.4%
CS5	55	21.8%
CS6	33	13.1%

**Table 3:** Distribution of the patients according to their gender and CVM stage.

Gender/CVM Stage	Girls		Boys		*p* *
	No.	Percentage	No.	Percentage	
CS1	21	11.80%	17	23%	0.001
CS2	14	7.90%	13	17.60%	
CS3	27	15.20%	8	10.80%	
CS4	41	23%	23	31.10%	
CS5	49	27.50%	6	8.10%	
CS6	26	14.60%	7	9.50%	

* Fisher´s Exact Test.

**Table 4:** Comparison of the chronological age in different CVM stages.

CVM Stage	Mean Age (Years) ± SD	Median (IQR)	Medium Rank	*p* *
CS1 (*p* = 0.752 **)	9.055 ± 1.51	8.9 (7.87–10.15)	48.62	<0.001
CS2 (*p* = 0.173 **)	10.11 ± 1.59	10.1 (8.8–11.6)	77.8	
CS3 (*p* = 0.700 **)	10.35 ± 1.75	10.2 (9–11.4)	84.99	
CS4 (*p* = 0.568 **)	12.05 ± 1.73	12.05 (11–12.9)	142.62	
CS5 (*p* = 0.048 **)	12.7 ± 1.72	12.9 (11.6–13.9)	166.39	
CS6 (*p* = 0.002 **)	13.76 ± 1.35	14.1 (13.2–14.65)	202.30	

SD, standard deviation; IQR, interquartile range; * Kruskal–Wallis H Test; ** Shapiro–Wilk Test.

**Table 5:** Post-hoc comparison of the chronological age in different CVM stages.

CVM Stage *	CS1	CS2	CS3	CS4	CS5	CS6
CS1	-	1.000	0.498	<0.001	<0.001	<0.001
CS2	1.000	-	1.000	0.002	<0.001	<0.001
CS3	0.498	1.000	-	0.003	<0.001	<0.001
CS4	<0.001	0.002	0.003	-	1.000	0.002
CS5	<0.001	<0.001	<0.001	1.000	-	0.378
CS6	<0.001	<0.001	<0.001	0.001	0.378	-

* Dunn–Bonferroni Post-Hoc Test.

**Table 6:** Comparison of the chronological age in different CVM stages for girls.

CVM Stage	Mean Age (Years) ± SD	Median (IQR)	Medium Rank	*p* *
CS1 (*p* = 0.908 **)	8.79 ± 1.56	8.7 (7.8–10)	29.38	<0.001
CS2 (*p* = 0.506 **)	9.49 ± 1.59	9.6 (8.25–11.1)	41.36	
CS3 (*p* = 0.489 **)	10.16 ± 1.69	10.1 (9–11.3)	54.59	
CS4 (*p* = 0.553 **)	11.99 ± 1.42	12 (10.95–12.75)	98.00	
CS5 (*p* = 0.076 **)	12.57 ± 1.74	12.9 (11.5–13.7)	114.53	
CS6 (*p* = 0.007 **)	13.58 ± 1.41	14 (13.07–14.6)	139.69	

SD, standard deviation; IQR, interquartile range; * Kruskal–Wallis H Test; ** Shapiro–Wilk Test.

**Table 7:** Post-hoc comparison of the chronological age in different CVM stages for girls.

CVM Stage *	CS1	CS2	CS3	CS4	CS5	CS6
CS1	-	1.000	1.000	<0.001	<0.001	<0.001
CS2	1.000	-	1.000	0.006	<0.001	<0.001
CS3	1.000	1.000	-	0.010	<0.001	<0.001
CS4	<0.001	0.006	0.010	-	1.000	0.019
CS5	<0.001	<0.001	<0.001	1.000	-	0.662
CS6	<0.001	<0.001	<0.001	0.019	0.662	-

* Dunn–Bonferroni Post-Hoc Test.

**Table 8:** Comparison of the chronological age in different CVM stages for boys.

CVM Stage	Mean Age (Years) ± SD	Median (IQR)	*p* * (*p* = 0.080 ***)
CS1 (*p* = 0.215 **)	9.38 ± 1.435	9.2 (7.85–10.75)	<0.001
CS2 (*p* = 0.162 **)	10.77 ± 1.354	11.5 (9.55–11.7)	
CS3 (*p* = 0.494 **)	11.01 ± 1.9	11.5 (8.9–12.3)	
CS4 (*p* = 0.398 **)	12.15 ± 2.215	12.4 (11–14)	
CS5 (*p* = 0.092 **)	13.76 ± 1.134	14 (13.17–14.57)	
CS6 (*p* = 0.792 **)	14.42 ± 0.838	14.6 (13.7–15)	

SD, standard deviation; IQR, interquartile range; * One-Way ANOVA Test; ** Shapiro–Wilk Test; *** Levene´s Test for homogeneity of variances.

**Table 9:** Post-hoc comparison of the chronological age in different CVM stages for boys.

CVM Stage *	CS1	CS2	CS3	CS4	CS5	CS6
CS1	-	0.246	0.241	<0.001	<0.001	<0.001
CS2	0.246	-	1.000	0.199	0.009	<0.001
CS3	0.241	1.000	-	0.582	0.044	0.003
CS4	<0.001	0.199	0.582	-	0.324	0.034
CS5	<0.001	0.009	0.044	0.324	-	0.982
CS6	<0.001	<0.001	0.003	0.034	0.982	-

* Tukey HSD Post-Hoc Test.

## Data Availability

The data presented in this study are available on request from the corresponding author. The data are not publicly available due to privacy reasons.
